# Racial trauma and its health impacts in Black people in the United States and Canada: a scoping review on conceptualizations, definitions and manifestations

**DOI:** 10.1186/s12889-025-25382-5

**Published:** 2025-11-19

**Authors:** Florence Kurai Mudzongo, Jessi Robinson, Jacob Albin Korem Alhassan

**Affiliations:** 1 College of Graduate and Postdoctoral Studies, Interdisciplinary Studies, University of Saskatchewan, Saskatoon, Canada; 2 Health Sciences Library, University of Saskatchewan, Saskatoon, Canada; 3 Department of Community Health and Epidemiology, College of Medicine, University of Saskatchewan, Saskatoon, Canada

**Keywords:** Racial Trauma, Race Based Traumatic Stress, Systemic Racism, Conceptualizations, Manifestations, Health Impacts, Black Communities, Scoping Review

## Abstract

Despite growing recognition of racism as a public health crisis, racial trauma remains inconsistently defined and under researched. This scoping review investigates how racial trauma is conceptualized in empirical literature, focusing on its manifestations and long-term health impacts among Black individuals and communities in the United States and Canada. Guided by the Joanna Briggs Institute methodology for conducting scoping reviews, we systematically searched ten multidisciplinary databases and identified 29 empirical studies published between 2000 and 2025. Most included studies were conducted in the United States of America, with only two studies that included Canadian participants. We find that racial trauma is described using varied terminology, such as race-based traumatic stress or race-related stress and arises from both direct and indirect exposure to racism. While some manifestations resemble post-traumatic stress disorder, racial trauma is a distinct trauma phenomenon shaped by chronic, cumulative, and systemic racism. It causes multidimensional harm, is experienced both individually and collectively, and may be transmitted intergenerationally even to neonates. This review identifies psychological, physiological, social and community-level impacts. These findings underscore the need for a unified conceptual framework, formal recognition of racial trauma in the Diagnostic and Statistical Manual of Mental Disorders, Fifth Edition, Text Revision (DSM-5-TR) and increased empirical research across contexts including in Canada. These findings have important implications for mental health policy and clinical practice, highlighting the need for culturally responsive frameworks that address the unique experiences of racial trauma among Black communities.

**Supplementary Information:**

The online version contains supplementary material available at 10.1186/s12889-025-25382-5.

## Introduction

Racial trauma is increasingly recognized as a critical public health and mental health concern, particularly for Black communities in Canada and the United States of America. In the Canadian context, scholars [1] have highlighted how systemic racism is traumatizing and impacts the health of Black populations. In the United States of America as well, racial trauma has been framed as both a public health crisis [2] and a reproductive justice issue [3], underscoring the urgency of addressing it through systemic, clinical, and policy interventions.

Racial trauma has garnered growing scholarly interest across diverse fields including psychology [4, 5], social work and pediatrics [6], trauma and acute surgery [7],and human development and family studies. Despite this interdisciplinary engagement, there remains no universally accepted definition of racial trauma. The term has been conceptualized in various ways, with some scholars emphasizing its historical and intergenerational dimensions [8, 9],and others highlighting how racism’s effects are transmitted through family narratives and embodied stress responses. Others focus on its systemic and institutional aspects, particularly within educational and health care settings [10–12]. Additionally, racial trauma is sometimes framed as a psychological and emotional injury from chronic exposure to racism [13–15]. However, this framing sometimes overlooks the physiological health consequences [9] as well as broader social and community-level impacts [11]. There are multiple overlapping terms used to describe racial trauma such as race-based stress [7], race-based trauma [16], and racism-related stress [17], highlighting the evolving, and interdisciplinary nature of the construct. However, the literature reflects substantial variability in how racial trauma is defined and measured, with overlapping terms and inconsistent use of assessment tools. This conceptual ambiguity complicates synthesis and underscores the need for clearer distinctions between racial stress and trauma.

Most of the existing literature on racial trauma advocates for viewing racial trauma as a distinct form of trauma. While sharing some symptomatology with post-traumatic stress disorder (PTSD), it differs in its chronicity, cumulative and sociocultural context and may not meet traditional diagnostic criteria for PTSD [11–14, 18]. Arguably, the absence of racial trauma in the Diagnostic and Statistical Manual of Mental Disorders Five (DSM-5-TR) reflects a broader epistemic marginalization and limits the development of culturally responsive mental health care. Many scholars have therefore called for racial trauma to be formally recognized within the DSM-5-TR, citing its widespread psychological impact and the limitations of existing diagnostic criteria [2, 7, 14, 19]. Despite these ongoing debates there are no current syntheses to conceptually clarify what is meant by racial trauma in the literature and to identify its impact on the health of Black communities.

This scoping review maps existing empirical research on racial trauma, with a focus on the Black population. It aims to clarify how racial trauma is defined and conceptualized in empirical studies, including its manifestations and health impacts. By identifying key features, conceptual trends and gaps, the review lays groundwork for future diagnostic and therapeutic frameworks that are clinically rigorous, culturally affirming and grounded in health equity.

## Methods

This scoping review was conducted in accordance with the Joanna Briggs Institute (JBI) [20, 21] methodological framework for scoping reviews, which includes the following steps: (1) identifying the research question, (2) identifying relevant studies, (3) selecting studies, (4) charting the data, and (5) collating, summarizing, and reporting the results. These steps were operationalized through a registered protocol, a librarian-assisted search strategy, dual independent screening, and thematic categorization of findings. In line with JBI guidance, the inclusion criteria were structured using the Population, Concept and Context (PCC) framework: Population-Black individuals and communities in the United States and Canada; Concept-racial trauma and related constructs (e.g., race-based traumatic stress, race-related stress), including conceptualizations, manifestations, and health impacts; Context- empirical studies conducted in the United States and Canada across health, education, and social science disciplines.

This review is reported in alignment with the Preferred Reporting Items for Systemic reviews and Meta-Analyses extension for Scoping Reviews(PRISMA-ScR) [22] guidelines. The overarching aim of this review was to systematically map the extant empirical research on racial trauma, with a particular focus on its conceptualization, definitional boundaries, manifestations, and associated health impacts among Black people in the United States of America and Canada.

To establish a comprehensive search strategy, an initial exploratory search of APA PsycINFO and Medline was undertaken in May 2025. This preliminary phase identified relevant terminology and refined the search syntax. Subsequently, in collaboration with a research librarian affiliated with the University of Saskatchewan (JR), a final search strategy was developed to ensure methodological rigour and comprehensiveness. The final search was conducted across ten multidisciplinary databases by two authors (FM and JR) to ensure broad coverage across health, education, and social sciences: APA PsycINFO, Medline, Anthropology Plus, PTSDpubs, Public Health Database, Web of Science Core Collection, Scopus, Sociological Abstracts, CINAHL and ERIC (Ovid). The complete search strategy for all databases is provided in Supplementary File S1.

Studies were eligible for inclusion if published in English between 2000 and 2025 and if they constituted empirical research. Eligible studies were required to focus on racial trauma or its related synonyms, such as race-related stress, race-based trauma, and race-based traumatic-stress. The population/participants in the study had to include participants who identified as Black, African American, African Canadian, Afro-Canadian, Afro-Caribbean. These terms were applied across databases to identify studies that either focused on or included Black participants in the context of racial trauma. While the inclusion criterion allowed for studies with mixed samples, the search strategy prioritized studies that centered Black populations, rather than those that included Black participants incidentally within broader samples. In studies that included participants who identified as ‘mixed race’ or that included multiple racialized groups, inclusion was determined based on whether the study provided disaggregated data or specific insights related to Black participants. Only empirical studies conducted within Canada and the United Sates of America were considered. The search string included (‘racial trauma’ OR ‘race-related stress’ OR ‘race-based traumatic stress’ OR ‘racism-related stress’) AND (‘Black’ OR ‘African American’ OR Afro-Canadian’) AND (‘Concept* OR Defin*). The full search string across databases is presented in supplementary information. FM and JAKA reviewed identified records and any disagreements between the two reviewers regarding study inclusion were resolved through discussion and consensus meetings. Non peer reviewed records such as dissertations and book chapters were excluded. Additionally, non-empirical studies, purely theoretical discussion papers and studies that did not include Black participants or were not directly about racial trauma were excluded.

**Fig. 1:**
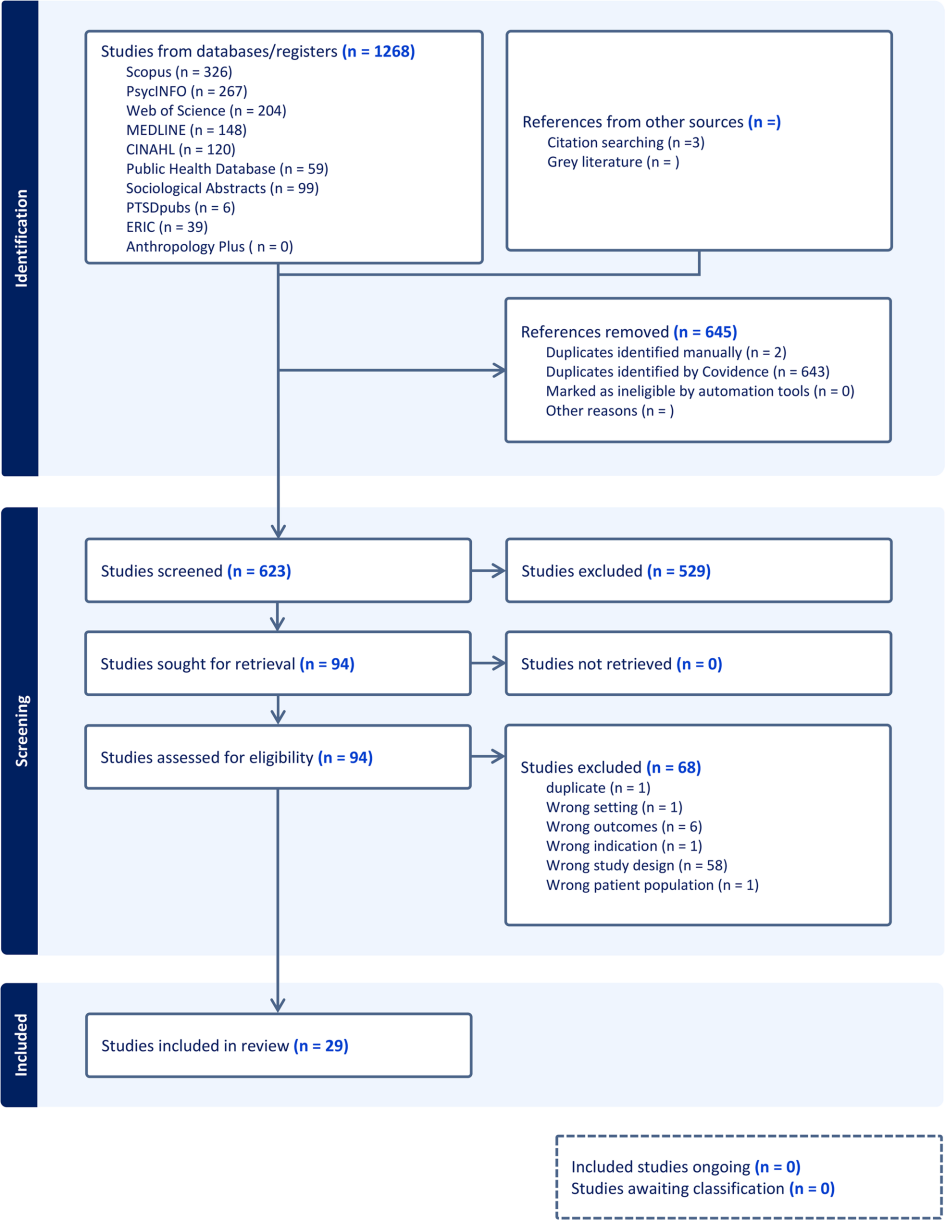
PRISMA diagram of included studies

The search findings from the database were exported to COVIDENCE software [23], followed by a systematic de-duplication and title/abstract screening which was conducted by two reviewers (FM and JAKA). The reference list of relevant articles was also searched to identify further studies which were reviewed to be added to the records. The relevance and appropriateness of articles selected for the final review were agreed upon by both reviewers. The scoping review protocol for this review was registered on the Open Science Framework (OSF): https://osf.io/urqnd/. This review did not receive any financial support from any funding agencies. A PRISMA-ScR diagram (Fig. 1) describing the literature search process and included studies is included below.

### Patient and public involvement

None.

## Findings

The systematic search of relevant databases resulted in a total of 1,268 records: Scopus (326), PsycINFO (267), Web of Science (204), Medline (148), CINAHL (120), Sociological Abstracts (99), Public Health (59), ERIC (39), PTSDpubs (6), and Anthropology (0). After removing 645 duplicates, 623 records were screened. Following title and abstract review, 529 records were excluded for not meeting the inclusion criteria. The full texts of 94 articles were assessed, and 68 were excluded for being non-empirical or not specifically focused on racial trauma. A total of 26 empirical studies met the inclusion criteria and were included in the final review, and 3 additional studies were identified through hand-searching and added to the final records, resulting in 29 included studies. Most studies adopted quantitative methodologies [2, 4, 5, 7, 9, 10, 12, 13, 17, 24–31] although many others were qualitative [8, 11, 15, 16, 18, 31, 32], and one study used mixed methods [14]. The participants in the included studies mainly identified as Black and or African American although one study included Black Caribbean participants [5], and another study included Black African and Afro-Caribbean participants [3]. This limited representation constrains the applicability of findings to the broader Black diaspora and underscores the need for more inclusive research that reflects diasporic diversity.

Once data charting was completed, we synthesized results by analyzing the explicit definitions that were provided in the articles, identified recurring key conceptual features of racial trauma in all the articles. We systematically identified the synonyms used to refer to racial trauma. Further, we synthesized how studies identified racial trauma manifestations, by categorizing them into 3 recurring sub-domains: psychological, physiological, and behavioral. We also synthesized the identified health impacts of racial trauma and categorized these into three sub-domains: psychological, physiological, and social/community impacts based on what was reported in the identified studies. While many studies identified specific psychological and physiological impacts others identified broader social and community level impacts experienced collectively and vicariously [11, 14, 26] hence our inclusion of social and community level impacts as a category. Based on the included studies, we distinguish the manifestations of racial trauma into acute and long-term chronic responses and health impacts. To ensure consistency, we categorized manifestations based on how they were framed in each study although some of the manifestations, such as insomnia, anxiety and depression may straddle both acute and chronic dimensions.

Given the diversity of theoretical frameworks and methodological approaches across the included studies, our categorization of psychological, behavioral, physiological, and social/community impacts reflects how these dimensions were framed in the original studies. While some overlap exists across categories, we aimed to preserve the integrity of each study’s conceptual framing while organizing findings thematically.

### Defining and conceptualizing racial trauma

Most studies [11, 12, 15, 18, 33] used the racial trauma definition from Comas-Díaz and colleagues [34]. This definition describes racial trauma as the psychological and emotional responses to real or perceived racial discrimination, including threats, humiliation, and witnessing racism, with ongoing individual and collective injuries due to repeated exposure to race-based stress. Other studies [8, 14, 18, 19, 35] used Carter’s [36], Race-Based Traumatic Stress model which conceptualizes racial trauma as emotionally painful, sudden, and overwhelming experiences from racism.

One study [8] cited the conceptual frameworks of Bryant-Davis & Ocampo [37] and Carter [36] to frame racial trauma as rooted in emotional pain and dehumanization, emphasizing its chronic and identity-threatening nature. Two studies [17, 27] drew on Harrell’s [38] Multilevel Model of Racism Related Stress to conceptualize racial trauma as a form of chronic, identity-threatening stress that arises when experiences of racism exceed individual or collective coping resources.

In contrast to studies that rely on previously established definitions, other scholars developed their own culturally grounded and context-specific conceptualizations. One study [16] defined it as a form of race-based stress resulting from inferior societal treatment, while another [5] framed it as cultural racism-related stress stemming from persistent devaluation of one’s racial group. In a third study [2], racial trauma was conceptualized through the lens of microaggressions as chronic, cumulative stressors. Notably one study [19] co-created an academic racial trauma definition with participants, identifying three defining features which are temporal (“sticking with”), intensity (“suffering severely”), and frequency (“repeating regularly”), to distinguish racial trauma from general race-related stress. This framing emphasizes that only particularly severe or repeated exposures to racism, especially in the absence of coping resources, results in racial trauma. A more recent study [18], expanded the conceptualization of racial trauma by co-constructing a definition with Black mental health professionals, grounded in clinical and lived experiences. Their definition emphasizes the cumulative, systemic, and embodied nature of racial trauma.

#### Key features of racial trauma

We identified 6 key features of racial trauma across the reviewed studies. First, racial trauma stems from direct or indirect exposure to racism. This exposure can be through interpersonal, institutional or cultural events [2, 12, 14, 15, 17, 30, 39]. Second, racial trauma is cumulative, chronic, and or acute. It builds over time through repeated exposure to racial stressors, while acute incidents such as police violence also contributes significantly to its development [4, 7, 10, 13, 14, 18]. Third, the harm caused by racial trauma is multidimensional, with immediate manifestations and long-term health impacts. Fourth, it can be intergenerational and transmitted in diverse ways, such as through families, community experiences, and media exposure [8, 9, 18, 28, 31, 35]. Fifth, racial trauma is systemic and identity-based, embedded within institutional, structural, and cultural systems that marginalize racialized people [11, 15, 16, 18, 19]. Finally, racial trauma is experienced both individually and collectively. Although some studies focused on individual experiences [7, 10, 13, 30], many others highlight its impact on families [3, 8, 14, 26, 32, 39], communities or group experiences [5, 19, 24].

**Table 1:** Overview of methodological and conceptual features identified across reviewed studies on racial trauma

Author (S)	Methodology			Definitions and Conceptualization						Manifestations			Health Impacts		
	Quant	Qual	Mix	Exposure to Racism direct/indirect	Cumulative, Chronic, and/or Acute	Multidimensional Harm	Intergenerational & Vicarious	Systemic & Identity-Based	Individual (x)- or Collective Experience-(xx)	Psychological	Physiological	Behavioral	Psychological	Physiological	Social & Community
Bentley-Edwards 2016 [24]	x			x	x	x		x	xx	x		x	x		x
Bird et al. 2021 [7]	x			x	x	x		x	x	x			x	x	
Brantley, 2023 [39]		x		x	x	x	x	x	xx	x	x	x	x	x	x
Brown et al. 2025 [18]		x		x	x	x	x	x	xx	x	x	x	x	x	x
Carter et al. 2013 [13]	x			x	x	x		x	x	x	x	x	x	x	
Carter and Reynolds, 2011 [25]	x			x	x	x		x	x	x	x		x	x	
Case and Hunter, 2014	x			x	x	x		x	x	x			x		
Dilworth-Bart et al. 2022 [32]		x		x	x	x	x	x	xx	x		x	x		x
Douglas et al. 2025 [8]		x		x	x	x	x	x	xx	x		x	x		x
Francois et al. 2024 [12]	x			x	x	x		x	x	x	x	x	x	x	x
Galan et al. 2024 [30]	x			x	x	x		x	x	x			x		x
Gaylord-Harden and Cunningham 2009 [17]	x			x	x	x		x	x	x		x	x		x
Gomez et al. 2023			x	x	x	x		x	xx	x	x	x	x		x
Greer, 2021	x			x	x	x		x	x	x			x		x
Gregory Jr. and Tucker Edmonds, 2024 [26]	x			x	x	x	x	x	x	x	x	x	x	x	x
Grier-Reed et al. 2021 [11]		x		x	x	x	x	x	xx	x		x	x	x	x
Hargons et al. 2022 [19]		x		x	x	x	x	x	xx	x	x	x	x	x	x
Leath et al. 2022 [3]		x		x	x	x	x	x	xx	x	x	x	x	x	x
McNeil-Young et al. 2023		x		x	x	x	x	x	x	x	x		x		x
Obenauf et al. 2023 [10]	x			x	x	x	x	x	x	x			x		
Odafe et al. 2017 [18]	x			x	x	x		x	xx	x			x		x
Kral et al. 2024 [9]	x			x	x	x	x	x	xx		x			x	
Smith Lee and Robinson, 2019 [35]		x		x	x	x	x	x	x	x	x	x	x	x	x
Williams, 2021 [15]		x		x	x	x	x	x	xx	x	x	x	x	x	x
Wyatt et al. 2021 [16]		x		x	x	x	x	x	xx	x		x	x		x
Williams & Zare, 2022 [4]	x			x	x	x		x	xx	x	x	x	x	x	
Roberson & Carter, 2022 [28]	x			x	x	x		x	x	x	x	x	x	x	
Tausen et al. (2023) [29]	x			x	x	x	x	x	xx	x		x	x		x
Zapolski et al. 2023 [2]	x			x	x	x		x	x	x		x	x		

#### Assessment tools and conceptual frameworks

The reviewed studies employed a range of standardized assessment tools and critical frameworks to measure racial trauma. Quantitative measures included the Index of Race-Related Stress–Brief (IRRS-B) [10, 25, 27], Race-Based Traumatic Stress Symptom Scale (RBTSS) [13, 14, 29], and the University of Connecticut Racial/Ethnic Stress & Trauma Survey (UnRESTS), which was developed to assess racial trauma within the DSM-5-TRPTSD symptom cluster [4]. Other tools used were the Perceived Ethnic Discrimination Questionnaire (PEDQ) [7], Schedule of Racist Events (SRE) [31], Racism Experience Questionnaire (REQ) [9], Cultural Trauma Scale (CuTS) [26], Adolescent Discrimination Distress Index (ADDI) [30], Online Victimization Scale [30], and the Racial and Ethnic Microaggressions Scale (REMS) [17]. It is important to note that while tools such as IRRS-B, REMS, and PEDQ are widely used, they primarily assess racial stress exposure rather than trauma-specific symptomatology, which may limit the precision of conclusions regarding racial trauma and its health impacts.

Qualitative studies often drew on critical and interpretive frameworks to guide their conceptualization of racial trauma. These included Critical Race Theory [16, 35], Black Feminist Qualitative Framework [19], the Constructivist-Interpretivist Paradigm [19], the RECAST model [3], and Nayak’s Black Feminist Framework.

#### Terminological variability and synonyms

Across the included studies terms such as racial trauma, race-based traumatic stress and race-related stress were frequently used interchangeably. In one study [12] authors explicitly stated that these terms including ethno-racial trauma are used interchangeably drawing on the literature and based on the work of foundational scholars like Carter [36] and Bryant-Davis & Ocampo [37]. Similarly another study [10] used terms such as race-related stress, racism-related stress, cultural race-related stress, and racism-related PTSD symptoms. A notable exception was in a study by Hargons and colleagues [19]. While the participants in their study used racial trauma and race-related stress interchangeably, the scholars’ thematic analysis revealed a critical conceptual distinction that not all race-based stressors result in racial trauma. Instead, racial trauma was understood to emerge when race related stressors are intense, frequent, unresolved due to limited coping or healing resources. Some of the most common terms used in the included studies were racism-related stress [17], race-based trauma [16], various racial trauma [3], race-related stress [27], cultural racism-related stress [5], and various racism-related stress [3, 39]. 

### Racial trauma manifestations

We synthesized and reported on racial trauma manifestations and impacts using categories such as psychological, behavioral, physiological, and social/community impacts based on the key categories often reported in the included studies. For example, in studies where the focus was on emotional stress and cognitive distortions, we grouped such manifestations under the category of psychological. In others where the impacts were primarily describing behaviours such as hypervigilance, substance use and others, we grouped these under behavioural impacts. In some cases the manifestations were purely physical such as hypertension, blood pressure and others and we therefore grouped these under physiological manifestations. Where impacts moved beyond the individual to relational and communal manifestations, we grouped these under the category of community and social impacts. In the sections that follow we describe the manifestations and health impacts of racial trauma based on these categories.

#### Psychological manifestations

Findings across the reviewed studies consistently identified psychological manifestations as the most immediate and pervasive response to racial trauma (see Table 1). These manifestations span a wide range of emotional, cognitive, and identity-related symptoms, often emerging in response to both direct and various racial harm. The most frequently reported psychological manifestations include various forms of emotional distress, anxiety, depressive symptoms, sadness, anger, confusion, fear, hopelessness, shame and grief [2–5, 10, 12, 13, 15–18, 26, 29, 30, 32, 39]. These manifestations were often described as persistent, overwhelming, and reactivated by contemporary racial violence or media exposure [15, 19, 33]. 

Cognitive distortions were common and included intrusive thoughts, rumination, dissociation, depersonalization, derealization, disbelief, and altered perception of safety or worldview [4, 15, 25, 27, 28]. Identity-related impacts were also prominent, such as low-self-esteem, self-doubt, insecurity about racial identity, internalized oppression, and diminished academic efficacy [10, 14, 19, 24, 27, 28, 31, 32]. These were often compounded by racial battle fatigue and emotional toll of unacknowledged suffering, especially in professional and academic settings [11, 12, 16, 18, 35]. 

#### Behavioral manifestations

Behavioural manifestations were identified in several of the reviewed studies, illustrating how Black individuals and communities respond to racial trauma through action and adaption. For a full list of sources, see Table 1. Common behaviours included avoidance of the police, predominantly white spaces, or media content related to racial violence [3, 14, 15, 19, 29, 32, 35, 39]. Suicidality was reported as a self-destructive response to overwhelming race-related stress [27]. Other responses included social withdrawal, emotional suppression, and overcompensation in professional or academic settings [8, 13, 16, 19]. Substance use, particularly alcohol and cannabis, was identified as a coping response to racial microaggressions and chronic stress [2]. Parenting behaviours such as hypervigilance, racial socialization, and protective monitoring were reported among Black mothers and fathers [3, 32, 39]. Additionally, social justice activism, protest, and creative expression (e.g. poetry), mentoring were described as forms of resistance [12, 33]. 

#### Physiological manifestations

Physiological manifestations were among the least frequently reported across the reviewed studies, yet those that reported them identified consistent patterns of somatic and stress related responses. The somatic manifestations included headaches, fatigue, gastrointestinal issues, trembling, heart palpitations, sweaty palms, and muscle tension [3, 4, 16, 17, 25, 33, 39, 40]. Stress related physical manifestations reported included cardiovascular strain, hypertension, sleep.

disturbance [13, 16, 17, 19, 25, 29, 31, 39]. One study [18] offered a qualitative insight from Black mental health professionals, describing clients who presented with chronic exhaustion, somatic pain, disrupted sleep, and physical tension. Additionally, physical reactions such as agitation, affective arousal were reported in response to intense racial stressors [19], while crying, heart racing and physiological discomfort were documented following exposure to traumatic media content related to racism and racial violence [15]. 

#### The unique nature of racial trauma

The reviewed studies consistently identified racial trauma as distinct from PTSD, despite overlapping manifestations such as hypervigilance, avoidance, and dissociation [2, 4, 7, 11, 14, 19, 28, 35]. Unlike PTSD, which is typically linked to discrete, life-threatening events (DSM-5-TR Criterion A), racial trauma is rooted in cumulative and systemic experiences of racism, including microaggressions and vicarious racial violence [2, 7, 9, 14]. One study [30] compared racial trauma to interpersonal trauma (e.g., physical assault), non-interpersonal trauma (e.g., natural disasters) and complex trauma. It concluded that racial trauma most closely resembles complex trauma due to its chronic, pervasive and identity-based nature. Moreover, the study [30] found that racial trauma was more strongly associated with PTSD, Major depressive disorder (MDD), and generalized anxiety disorder GAD than the other trauma types.

### Racial trauma health impacts

#### Psychological impacts

While racial trauma is itself a consequence of exposure to racism, findings across the reviewed studies indicate that it also contributes to long-term deterioration of psychological health. These impacts extend beyond immediate emotional or cognitive responses and include diagnosable mental health conditions, such as clinical depression [2, 10, 12–14, 27, 29–31, 31, 35], and anxiety disorders [9, 15–17, 19, 39]. PTSD and increased vulnerability to trauma related disorders were reported in several studies [3, 7, 10, 19, 29, 30, 35]. These studies [7, 14, 19, 30, 35] explicitly distinguished racial trauma from PTSD. Other mental health impacts reported in the reviewed studies included chronic stress and emotional dysregulation [10, 13, 14, 19, 31]. 

Several studies described the psychological toll of racial trauma as enduring, highlighting the cumulative and intergenerational transmission of psychological distress [8, 15, 32, 33, 39]. Others also emphasized vicarious trauma through media exposure and storytelling which simultaneously acted as a mechanism of harm and healing [8, 14, 15, 19, 29, 32]. Long term grief was reported in response to racial violence and loss [16, 35]. Developmental and lifespan psychological impacts reported in some studies include disruption of child and adolescent development involving emotional dysregulation and identity disturbance [17, 30, 32]. One study [9] reported impacts on neonates, finding altered connectivity of the amygdala and hippocampus in infants exposed to maternal racial stress, suggesting a predisposition to anxiety and depression. Existential despair involving loss of meaning, purpose, feelings of emptiness and lack of motivation was also reported [4, 40]. 

#### Physiological impacts

In contrast to acute physiological manifestations, several studies identified long-term health consequences of chronic exposure to racial trauma. These included elevated risk of hypertension and cardiovascular disease [17, 19, 25, 31]. Racial trauma was also linked to early mortality and physiological deterioration, particularly among Black women, where sustained exposure to racism was associated with weathering and increased maternal health risks [19]. 

#### Social and community impacts

Racial trauma extends beyond the individual, disrupting social cohesion and community wellbeing through vicarious exposure to racial violence, systemic oppression, and intergenerational stress [7, 12, 15, 29, 35]. Common impacts included social withdrawal, alienation, and strained interpersonal relationships [15, 16, 30, 31, 39]. Disrupted relationships were especially evident in academic and family contexts, where participants reported exclusion, cultural invalidation, and interpersonal strain [8, 12, 16, 31]. In some studies, participants expressed distrust, particularly toward institutions and law enforcement, shaped by repeated exposure to racial violence and systemic neglect [14, 15, 35]. Community mourning and grief were recurring responses to racialized events, often accompanied by heightened concern for younger generations [3, 8, 19]. 

Additional social impacts included strained professional relationships, and emotional labour, among Black professionals in predominately white spaces [16, 18]. Disconnection from cultural identity and reduced quality of life were also noted [5, 24, 32]. 

#### Strength-based and adaptive social and community responses to racial trauma

Despite the harms of racial trauma, Black communities demonstrated strength-based and adaptive responses rooted in resilience, cultural identity, and collective care. These included protective parenting strategies (e.g., racial socialization, media monitoring and, racial armouring to shield children from racial harm) to foster empowerment [3, 32]. Storytelling and intergenerational knowledge sharing were used to strengthen family bonds and promote healing [8, 33]. Social justice activism, protest, and civic engagement were also reported as healing community responses to racial trauma, offering expression, agency, resistance, and solidarity [15, 18, 33]. 

## Discussion

This scoping review synthesized 29 empirical studies on racial trauma, revealing conceptual richness but methodological fragmentation in how it is defined, assessed, and studied in Black communities. Foundational scholars such as Carter [36], Bryant-Davis and Ocampo [37], and Comas-Díaz and collegues [34] frame racial trauma as cumulative, systemic, and comparable to other forms of interpersonal violence. Their work positions racial trauma as a multidimensional phenomenon rooted in emotional pain, identity threat, and collective harm. Yet, many of the reviewed studies [2, 5, 10–12, 24, 26, 27, 30, 31] cite these frameworks without necessarily fully engaging their clinical implications or operationalizing them in study design. This pattern reflects a reliance on racial trauma theory without corresponding methodological or diagnostic rigor. As a result, the field lacks standardized approaches to assessing racial trauma and translating its theoretical complexity into clinical practice.

While several studies [7, 8, 15, 35] reference Bryant-Davis and Ocampo’s [37] foundational work to conceptualize racial trauma, only a few studies [13, 14, 26, 33] engage with their analogy between racist incidents and interpersonal violence such as sexual assault and domestic abuse. This analogy challenges narrow, event-based definitions of trauma and calls for frameworks that recognize racial trauma as emotionally and psychologically injurious, even without physical harm. While the studies [7, 8, 15, 35] acknowledge the chronic and systemic nature of racial trauma, they largely stop at conceptual framing and do not translate the analogy into methodological design, assessment strategies, or clinical practice implications. In contrast, one study [13] developed the Race-Based Traumatic Stress Symptom Scale (RBTSSS); another study [14] integrated Black feminist and liberation psychology into a healing framework; a third study [26] created the Cultural Trauma Scale (CuTS) and another study [33] used qualitative methods to explore storytelling and activism as therapeutic responses. These studies exemplify how the analogy can inform both theory and practice, offering models for trauma-informed care that reflect the lived realities of racialized individuals.

Intergenerational dimension of racial trauma remains underexplored. Although many studies [7–9, 14–16, 32, 33] acknowledge multigenerational nature, few investigate mechanisms through which it is transmitted across generations. For instance, one study [9] offered compelling physiological evidence of intergenerational transmission by linking maternal racial stress during pregnancy to altered neonatal brain development, highlighting biological pathways such as heightened glucocorticoid exposure. Similarly, these studies [7, 16] move beyond conceptual framing to explore how racial trauma is carried through storytelling, silence, and vicarious identification with community suffering. Studies [8, 14, 15, 32] further enrich this discourse by documenting how trauma is transmitted through family dynamics, maternal hypervigilance, and community-based coping strategies. While the intergenerational, collective, and systemic dimensions of racial trauma are conceptually compelling, more empirical research is needed to further understand how these operate. Many studies relied on data from small samples with only one offering physiological evidence linking maternal racial stress to neonatal brain development. There is the need for longitudinal designs, biological measures, and larger samples to more robustly examine intergenerational aspects of racial trauma. Similarly, evidence on collective and systemic impacts require large-scale empirical validation.

Despite the growing body of research, definitional clarity remains inconsistent. Terms such as race-based stress, race-related traumatic stress, racial trauma, and racial microaggressions are often used interchangeably [12, 34] reflecting the complexity of racialized harm but complicating measurement, intervention design, and cross-study comparability. The conflation of stress and trauma responses risks obscuring the depth and gravity of trauma-specific symptomatology. This variability also introduces interpretive subjectivity, particularly when synthesizing findings across studies that do not clearly distinguish between racial stress and racial trauma. Moreover, the lack of consistent criteria for categorizing acute versus chronic manifestations further complicates efforts to map the health impacts of racial trauma with precision. This conceptual slippage is evident in these studies [12, 24], which employ racial trauma terminology but rely on proxy measures that assess racial stress exposure rather than trauma manifestations. One study [12] used the Racial and Ethnic Microaggressions Scale (REMS) to quantify the frequency of racial microaggressions experienced by Black students in academic settings. While this assessment tool effectively captures exposure to racialized stressors, it does not assess the psychological or physiological symptoms associated with trauma. Similarly, another study [24] employed the Index of Race-Related Stress–Brief (IRRS-B), a validated measure of racism-related stress across cultural, institutional, and individual domains. However, the IRRS-B also focuses on stress exposure rather than trauma symptomatology. While valuable for capturing aspects of racialized stress, many commonly used measures such as the Racial and Ethnic Microaggressions Scale (REMS) [2, 12], the Index of Race-Related Stress–Brief (IRRS-B) [24, 27], and the Perceived Ethnic Discrimination Questionnaire (PEDQ) [7], lack the specificity needed to fully conceptualize and assess racial trauma as a distinct construct. These instruments often focus on exposure to racism or stress responses rather than trauma symptomatology. In contrast, tools such as the Race-Based Traumatic Stress Symptom Scale (RBTSSS) [13, 14] and UnRESTS [4] offer more targeted assessments, yet remain underutilized. As such, conclusions drawn from these studies regarding racial trauma’s health impacts should be interpreted with caution, particularly when based on instruments that assess racial stress exposure rather than trauma-specific symptomatology.

In contrast, one qualitative study [19] underscored the importance of definitional precision through participant-driven insights. Black students at a predominantly White institution differentiated race-based stress from racial trauma using three experiential components: temporal duration, intensity of impact, and frequency of exposure. Their narratives revealed that while racial stress may be acute and situational, racial trauma is often chronic, cumulative, and deeply embedded in one’s sense of self and safety. This experiential clarity challenges the fluid use of terminology and highlights the need for frameworks that honour the lived complexity of racial harm.

Other dimensions of racial trauma might be better understood through the incorporation of the life-course framework that captures the cumulative, systemic, and intergenerational nature of racial trauma [41]. Additionally, in contexts such as Canada where the dominant discourse emphasizes multiculturalism [1] there is a propensity to obscure systemic racism and racial trauma experienced by Black, Indigenous, and other racialized communities. Some emerging literature [42] from the United States of America, proposes cultural humility as a foundation for healing ethno-racial trauma, a term that foregrounds the ethnic dimension often overlooked in standalone notions of racial trauma.

The reviewed literature highlights that racial trauma is a complex and multidimensional phenomenon characterized by overlapping features such as chronic exposure to racism, cumulative stress, systemic and interpersonal discrimination, and identity-based harm. While the definitional diversity across studies presents challenges for synthesis, it also reflects the evolving and interdisciplinary nature of the construct. More literature and data are needed to further explore how racial trauma intersects with other vulnerabilities to produce poor health.

Distinguishing between manifestations and health impacts offers a critical framework for understanding the layered and cyclical nature of racial trauma. This separation moves beyond symptom cataloging to illuminate how racial trauma operates as both an immediate disruption and a long-term condition shaped by systemic forces. Acute manifestations such as hypervigilance, emotional exhaustion, and sleep disturbances are not simply psychological reactions; they are embodied signals of ongoing racialized stress rooted in historical and institutional violence [16, 19, 39]. Though these manifestations resemble PTSD symptoms, their origins lie in chronic exposure to racism rather than discrete traumatic events [7, 13, 14, 19, 30]. Given the heterogeneity of study designs and conceptual frameworks, our synthesis of racial trauma impacts necessarily involved interpretive judgment. We acknowledge that the categorization into psychological, behavioral, physiological, and social/community domains is a thematic mapping rather than a standardized classification. While we aimed to remain consistent with how each study presented its findings, the lack of uniform criteria across the literature introduces a degree of subjectivity. These categories should therefore be viewed as heuristic tools for organizing the literature and identifying patterns, rather than definitive constructs.

Rather than viewing racial trauma manifestations as isolated or transient, it is essential to recognize their role in initiating long-term health deterioration. Racial trauma not only emerges from systemic racism but also acts as a catalytic condition that worsens vulnerability to a range of psychological and physiological disorders. It rarely presents as a standalone diagnosis instead, it co-occurs with and intensifies conditions such as depression, anxiety, suicidality, and PTSD-like symptoms [7, 9, 13, 27, 30, 31, 35]. This syndemic pattern of distress is further evidenced by physiological impacts including elevated blood pressure [31], somatic complaints [39] and disruptions in neonatal brain development [9], underscoring the intergenerational transmission of trauma through biological pathways. Behavioral manifestations such as substance misuse [2] reflect coping responses to unaddressed trauma, underscoring the need for trauma frameworks that are culturally responsive, intersectional, and attuned to the multidimensionality of racialized harm.

Crucially, the findings illuminate how racial trauma exceeds the boundaries of individual pathology, revealing its deeply collective and systemic nature. Collective grief, social withdrawal, and institutional distrust [11, 15, 35] signals that racial trauma is not merely experienced in isolation but is experienced collectively across social networks and cultural memory. These communal manifestations disrupt dominant trauma paradigms that center clinical symptomatology and individual resilience, exposing their limitations in capturing the full scope of racialized harm. Instead, the findings call for interpretive frameworks that foreground historical continuity, structural violence, and cultural specificity. This shift from individual to collective analysis invites a reimagining of trauma-informed care, one that is attuned to the sociopolitical contexts in which trauma unfolds and the communal strategies of survival and resistance that emerge in response.

Recognition of racial trauma as a multidimensional phenomenon also requires interrogating research practices that may inadvertently obscure the diversity within Black communities. A critical gap lies in the homogenization of Black participants, where “Black” is often used as a catch-all category, obscuring the complex and intersectional realities of diasporic Black communities. This lack of specificity limits the generalizability of findings and risks reinforcing the erasure of ethno-racialized experiences that are intertwined with racial trauma. Some studies [5, 43] offer compelling critiques of this tendency, pointing to the distinct experiences of Black Caribbean immigrants and the failure of Canadian research to account for ethnic, linguistic, and immigration-based differences. Yet these insights remain underutilized in study design and analysis. Comas-Díaz and colleagues [34] emphasize that racial trauma must be understood through an intersectional lens that accounts for the interlocking systems of oppression including race, gender, sexual orientation, and xenophobia that shape the lived experiences of racialized individuals. To move beyond this limitation, future research should intentionally include diverse Black populations and disaggregate data to reflect the nuances of racial trauma across ethnic, cultural, and migration histories. The underrepresentation of Black Caribbean, African, and Afro-Caribbean communities not only restricts the scope of current findings but also risks reinforcing dominant narratives that overlook the sociopolitical and transnational dimensions of racial trauma. Expanding participant diversity and contextual specificity will strengthen the empirical foundation for culturally responsive interventions and ensure that trauma-informed care reflects the lived realities of all Black communities.

While this review specifically focused on Black populations to address a significant gap in the literature, we acknowledge that racial trauma is a relational and systemic phenomenon that can affect individuals across racial groups. Future research would benefit from comparative studies that explore how racial trauma manifests across different racial communities to avoid reinforcing binary framings and to deepen understanding of its structural and interpersonal dimensions.

In sum, greater definitional rigor is essential for accurately capturing the lived realities of racialized individuals and communities, guiding targeted interventions, and advancing culturally grounded understandings of racial harm and healing.

## Strengths and limitations

A key strength of this scoping review is its comprehensive and interdisciplinary approach to the mapping and conceptualization, manifestations, and health impacts of racial trauma. By including quantitative, qualitative, and mixed studies, as well as diverse theoretical frameworks, the review captures the complexity and evolving nature of the construct. The inclusion of studies using synonymous or adjacent terms (e.g. race-based traumatic stress, vicarious racial trauma, and race-related stress) allowed for a broader and more inclusive synthesis of the literature.

However, several limitations should be noted. First, the review was limited to studies published in English and conducted primarily in North America, potentially excluding many relevant perspectives from Black populations globally. Second, while the inclusion of diverse terminology enriched the conceptual mapping, it also introduced challenges in comparing findings due to inconsistent definitions and measurement tools. Finally, the exclusion of grey literature, clinical/counseling book chapters, non-empirical studies and dissertations may have omitted valuable insights from community organizations, emerging scholars, practitioner-oriented texts that often shape the discourse on racial trauma.

In addition, the included studies exhibit considerable methodological variation, with differences in design, sample size, and theoretical orientation that complicate direct comparison and synthesis. The use of overlapping terminology such as racial trauma, race-based stress, and race-related traumatic stress contributes to conceptual ambiguity and limits the precision of thematic categorization. Participant representation was also uneven, with most studies focusing on African American populations and only a few including Black Caribbean, African, or Afro-Canadian participants. This restricts the generalizability of findings across the broader Black diaspora and may obscure important ethno-racial and cultural differences in experiences of racial trauma. Finally, although thematic synthesis was conducted with care, the diversity of conceptual frameworks required interpretive judgment in categorizing manifestations and health impacts. These categories should be viewed as heuristic tools for organizing the literature rather than definitive classifications. Future research would benefit from standardized trauma-specific measures, longitudinal designs, and the intentional inclusion of diverse Black populations to enhance empirical rigor and reduce potential subjectivity in interpretation.

## Conclusion

This scoping review reveals that racial trauma is a complex, evolving construct characterized by diverse definitions, manifestations, and health impacts. It is shaped by systemic, cumulative, identity-based, and intergenerational forces that distinguish it from conventional trauma such as may be experienced with PTSD. These findings underscore the need for culturally responsive, contextually grounded approaches to both research and care. While definitional diversity reflects the richness of lived experiences across diasporic Black communities, greater conceptual coherence is needed not to constrain this complexity, but to ensure it is meaningfully captured and applied. Clearer distinctions between stress and trauma are essential for accurately identifying racial harm, guiding targeted interventions, and advancing inclusive models of healing. The are several policy and practice implications from this study including the need for public health agencies to integrate racial trauma into health equity frameworks, recognizing its role in shaping mental and physical health disparities. This includes funding community-led healing initiatives, supporting intergenerational trauma recovery programs, and embedding racial trauma awareness into public health education, surveillance, and prevention strategies. Mental health, education, and policy sectors in the USA and Canada could also integrate trauma-specific assessment tools validated for Black populations and train clinicians in culturally responsive care.

## Supplementary Information

Supplementary Material 1.

## Data Availability

No datasets were generated or analysed during the current study.

## Abbreviations

PSTD: Post Traumatic Stress Disorder
RBTSSS: Race-Based Traumatic Stress Symptom Scale
MDD: Major depressive disorder
GAD: Generalized anxiety disorder
REMS: Racial and Ethnic Microaggressions Scale
IRRS-B: Index of Race-Related Stress–Brief
REMS: Racial and Ethnic Microaggressions Scale
PEDQ: Perceived Ethnic Discrimination Questionnaire
CuTS: Cultural Trauma Scale
DSM-5-TR: Diagnostic and Statistical Manual of Mental Disorders, Fifth Edition, Text Revision

## References

[1] Williams MT, Khanna Roy A, MacIntyre MP, Faber S. The traumatizing impact of racism in Canadians of colour. Curr Trauma Rep. 2022;8(2):17–34. 10.1007/s40719-022-00225-5. 35345606 10.1007/s40719-022-00225-5 PMC8943361

[2] Zapolski TCB, Rowe AT, Clifton RL, Khazvand S, Crichlow QJ, Faidley M. Examining the unique and additive effect of trauma and racial microaggressions on substance use risk among Black young adults. Cult Divers Ethn Minor Psychol. 2023;29(3):289–301. 10.1037/cdp0000480. 10.1037/cdp0000480 PMC9218005 34941280

[3] Leath S, Butler-Barnes S, Haynes-Thoby L. They just keep coming: a study of how anti-Black racial violence informs racial grief and resistance among Black mothers. J Child Fam Stud. 2022;31(12):3450–67. 10.1007/s10826-022-02421-y. 36105272 10.1007/s10826-022-02421-y PMC9461437

[4] Williams M, Zare M. A psychometric investigation of racial trauma symptoms using a semi-structured clinical interview with a trauma checklist (UnRESTS). Chronic Stress. 2022;6:24705470221145126. 10.1177/24705470221145126. 36578698 10.1177/24705470221145126 PMC9791291

[5] Case AD, Hunter CD. Cultural racism–related stress in Black Caribbean immigrants: examining the predictive roles of length of residence and racial identity. J Black Psychol. 2014;40(5):410–23. 10.1177/0095798413493926.

[6] Woolverton GA, Yip T, Rastogi R, Hahm HC, Liu CH. Differential associations between race-based traumatic stress and major, everyday, and vicarious racial discrimination among young adults of color. J Trauma Stress. 2025;38(2):330–40. Available from: https://onlinelibrary.wiley.com/10.1002/jts.23130 . Cited 2025 July 7. 10.1002/jts.23130 39907617

[7] Bird CM, Webb EK, Schramm AT, Torres L, Larson C, deRoon-Cassini TA. Racial discrimination is associated with acute posttraumatic stress symptoms and predicts future posttraumatic stress disorder symptom severity in trauma‐exposed Black adults in the United States. J Trauma Stress. 2021;34(5):995–1004. 10.1002/jts.22670. 33715212 10.1002/jts.22670 PMC9123835

[8] Douglas J, Perlstein M, Polanco-Roman L. Toward an understanding of intergenerational trauma and storytelling in Black families. Psychol Trauma Theory Res Pract Policy. 2025;17(2):256–63. 10.1037/tra0001746. 10.1037/tra0001746 38934938

[9] Kral TRA, Williams CY, Wylie AC, McLaughlin K, Stephens RL, Mills-Koonce WR et al. Intergenerational effects of racism on amygdala and hippocampus resting state functional connectivity. Sci Rep. 202;14(1):17034. Available from: https://www.nature.com/articles/s41598-024-66830-3. Cited 2025 July 10. 10.1038/s41598-024-66830-3 PMC11266580 39043776

[10] Obenauf C, Mekawi Y, Lathan EC, Hinojosa CA, Thomas JG, Stevens JS, et al. Indirect effect of race-related stress on traumatic stress and depression symptoms via subjective social status in a Black community sample. Am J Community Psychol. 2023;72(1–2):116–26. 10.1002/ajcp.12693. 37434412 10.1002/ajcp.12693

[11] Grier-Reed T, Said R, Quiñones M. From Antiblackness to Cultural Health in Higher Education. Educ Sci. 2021;11(2):57. Available from: https://www.mdpi.com/2227-7102/11/2/57. Cited 2025 July 10.

[12] Francois S, Blakey J, Stevenson R, Walker T, Davis C. Navigating COVID-19 and racial trauma as a Black student at predominantly White institutions. Am J Community Psychol. 2024;73(1–2):66–77. 10.1002/ajcp.12668. 37079437 10.1002/ajcp.12668

[13] Carter RT, Mazzula S, Victoria R, Vazquez R, Hall S, Smith S, et al. Initial development of the Race-Based Traumatic Stress Symptom Scale: assessing the emotional impact of racism. Psychol Trauma Theory Res Pract Policy. 2013;5(1):1–9. 10.1037/a0025911.

[14] Gomez J, Reid L, Polanco-Roman L, Barney A, Peyton C, Olugbemiga O. Self- and collective care as radical acts: a mixed-method study on racism-based traumatic stress among emerging adults. Am J Orthopsychiatry. 2024;94(1):61–76. 37768607 10.1037/ort0000705

[15] Williams S. Stream of sadness: young black women’s racial trauma, police brutality and social media. Fem Media Stud. 2021;21(8):1270–84. 10.1080/14680777.2021.2006261.

[16] Wyatt TR, Taylor TR, White D, Rockich-Winston N. When No One Sees You as Black: The Effect of Racial Violence on Black Trainees and Physicians. Acad Med. 2021;96(11S):S17–22. Available from: https://journals.lww.com/10.1097/ACM.0000000000004263. Cited 2025 July 10. 10.1097/ACM.0000000000004263 34348386

[17] Gaylord-Harden NK, Cunningham JA. The impact of racial discrimination and coping strategies on Internalizing symptoms in African American youth. J Youth Adolesc. 2009;38(4):532–43. 10.1007/s10964-008-9377-5. 19636726 10.1007/s10964-008-9377-5

[18] Brown EM, Cabell A, Gatabazi R, Gong J, Moran D, Sudan Z, et al. We do this till we heal us: Black mental health professionals’ experiences working with Black patients suffering from racial trauma. Psychother. 2025;62(2):154–63. 10.1037/pst0000554. 10.1037/pst0000554 39666471

[19] Hargons CN, Malone N, Montique C, Dogan J, Stuck J, Meiller C, et al. White people stress me out all the time: Black students define racial trauma. Cultur Divers Ethnic Minor Psychol. 2022;28(1):49–57. Available from: 10.1037/cdp0000351. Cited 2025 July 22. . 34291977 10.1037/cdp0000351 PMC8776568

[20] Peters MD, Godfrey C, McInerney P, Munn Z, Tricco AC, Khalil H. Scoping reviews. In: Aromataris E, Lockwood C, Porritt K, Pilla B, Jordan Z, editors. JBI Manual for Evidence Synthesis. JBI; 2024. Available from: https://jbi-global-wiki.refined.site/space/MANUAL/355862497/10.+Scoping+reviews. Cited 2025 May 28.

[21] Khalil H, Jia R, Moraes EB, Munn Z, Alexander L, Peters MDJ, et al. Scoping reviews and their role in identifying research priorities. J Clin Epidemiol. 2025;181:111712., 111712 Available from: https://linkinghub.elsevier.com/retrieve/pii/S0895435625000459. Cited 2025 May 28. 39924126 10.1016/j.jclinepi.2025.111712

[22] Tricco AC, Lillie E, Zarin W, O’Brien KK, Colquhoun H, Levac D, et al. PRISMA Extension for Scoping Reviews (PRISMA-ScR): Checklist and Explanation. Ann Intern Med. 2018;169(7):467–73. Available from: https://www.acpjournals.org/10.7326/M18-0850. Cited 2025 May 28 . 30178033 10.7326/M18-0850

[23] Kellermeyer L, Harnke B, Knight S. Covidence and Rayyan. J Med Libr Assoc. 2018;106(4). Available from: http://jmla.pitt.edu/ojs/jmla/article/view/513. Cited 2025 June 1.

[24] Bentley-Edwards KL. Hope, agency, or disconnect. J Black Psychol. 2016;42(1):73–99. 10.1177/0095798414557670.

[25] Carter RT, Reynolds AL. Race-related stress, racial identity status attitudes, and emotional reactions of black Americans. Cultur Divers Ethnic Minor Psychol. 2011;17(2):156–62. 21604839 10.1037/a0023358

[26] Gregory VL, Tucker Edmonds J. Cultural trauma scale: psychometric evaluation of Black men’s beliefs, emotions, and coping. Psychol Trauma Theory Res Pract Policy [Internet]. 2024;16(8):1329–37. 10.1037/tra0001607. 10.1037/tra0001607 37917451

[27] Odafe MO, Salami TK, Walker RL. Race-related stress and hopelessness in community-based African American adults: moderating role of social support. Cultur Divers Ethnic Minor Psychol. 2017;23(4):561–9. 10.1037/cdp0000167. 28604020 10.1037/cdp0000167

[28] Roberson K, Carter RT. The relationship between race-based traumatic stress and the trauma symptom checklist: does racial trauma differ in symptom presentation? Traumatology. 2022;28(1):120–8. 10.1037/trm0000306.

[29] Tausen BM, Misgano M, Wilson B. Campus Racial Climate, Psychological Well-being, and Race-Based Traumatic Stress Symptoms Among Monoracial Black and Biracial Black Students Following Heightened Exposure to Police Brutality. J Racial Ethn Health Disparities. 2024;11(1):121–31. 10.1007/s40615-022-01503-3. 36648621 10.1007/s40615-022-01503-3 PMC9844182

[30] Galán CA, Polanco-Roman L, Willis HA, Satinsky EN, Mateo Santana A, Ebrahimi CT et al. Is racism like other trauma exposures? Examining the unique mental health effects of racial/ethnic discrimination on posttraumatic stress disorder (PTSD), major depressive disorder (MDD), and generalized anxiety disorder (GAD). Am J Orthopsychiatry. 2024; Available from: 10.1037/ort0000807. Cited 2025 July 22. 10.1037/ort0000807 39509185

[31] Greer TM. The moderating role of coping strategies in Understanding the effects of Race-related stress on Academic Self-concept for African American Students. Psychology of Women Quarterly. 2011;35(2):215–26.

[32] Dilworth-Bart JE, Wallace B, Olaiya O. Black fathers’ personal histories, worldviews, and fathering behaviors. Fam Relat [Internet]. 2022;71(5):1896–916. 10.1111/fare.12631.

[33] McNeil-Young VA, Mosley DV, Bellamy P, Lewis A, Hernandez C. Storying survival: an approach to radical healing for the Black community. J Couns Psychol [Internet]. 2023;70(3):276–92. 10.1037/cou0000635. 37023277 10.1037/cou0000635

[34] Comas-Díaz L, Hall GN, Neville HA. Racial trauma: Theory, research, and healing: Introduction to the special issue. Am Psychol. 2019;74(1):1–5. Available from: 10.1037/amp0000442. Cited 2025 July 31. 30652895 10.1037/amp0000442

[35] Smith Lee JR, Robinson MA. That’s my number one fear in life. It’s the police: examining young Black men’s exposures to trauma and loss resulting from police violence and police killings. J Black Psychol [Internet]. 2019;45(3):143–84. 10.1177/0095798419865152.

[36] Carter RT. Racism and psychological and emotional injury. Couns Psychol. 2007;35(1):13–105. 10.1177/0011000006292033.

[37] Bryant-Davis T, Ocampo C. Racist incident–based trauma. Couns Psychol. 2005;33(4):479–500. 10.1177/0011000005276465.

[38] Harrell SP. A multidimensional conceptualization of racism-related stress: implications for the well-being of people of color. Am J Orthopsychiatry [Internet]. 2000;70(1):42–57. 10.1037/h0087722. 10702849 10.1037/h0087722

[39] Brantley M. Burdens of the what-if: Vicarious anti‐ Black racism and stress for Black mothers. J Marriage Fam. 2023;85(4):941–61. Available from: https://onlinelibrary.wiley.com/10.1111/jomf.12914. Cited 2025 July 8.

[40] Carter SE, Gibbons FX, Beach SRH. Measuring the biological embedding of racial trauma among Black Americans utilizing the RDoC approach. Dev Psychopathol. 2021;33(5):1849–63 Available from: https://www.cambridge.org/core/product/identifier/S0954579421001073/type/journal_article. Cited 2025 May 28. . 35586028 10.1017/s0954579421001073 PMC9109960

[41] Cénat JM. Complex racial trauma: evidence, theory, assessment, and treatment. Perspect Psychol Sci. 2023;18(3):675–87. 36288462 10.1177/17456916221120428 PMC10186562

[42] Akerele O, McCall M, Aragam G. Healing Ethno-Racial Trauma in Black Communities: Cultural Humility as a Driver of Innovation. JAMA Psychiatry. 2021;78(7):703, Available from: https://jamanetwork.com/journals/jamapsychiatry/fullarticle/2778478. Cited 2025 May 22. 33881471 10.1001/jamapsychiatry.2021.0537

[43] Williams KKA, Lofters A, Baidoobonso S, Leblanc I, Haggerty J, Adams AM. Embracing Black heterogeneity: the importance of intersectionality in research on anti-Black racism and health care equity in Canada. Can Med Assoc J. 2024;196(22):E767–9. Available from: http://www.cmaj.ca/lookup/10.1503/cmaj.230350. Cited 2025 Aug 3. 10.1503/cmaj.230350 PMC11173650 38857933

