# Supplementary material for: Racial trauma and its health impacts in Black people in the United States and Canada: a scoping review on conceptualizations, definitions and manifestations
Source: BMC Public Health. 2025 Nov 19;25:4034. doi: 10.1186/s12889-025-25382-5 (PMC12628536; doi:10.1186/s12889-025-25382-5)
Supplement: Supplementary file 1 — Supplementary Material 1. [file 12889_2025_25382_MOESM1_ESM.docx]

# Supporting information

S1 Supplementary file. This file contains the complete search strategies used across all databases for the scoping review.

S1 Fig 1. PRISMA-ScR flow diagram illustrating the selection process of studies included in the scoping review on racial trauma.

# S1Table 1. Overview of methodological and conceptual features identified across reviewed studies on racial trauma.

## S1 Supplementary file

Database Search Concepts Search String # of Results Date searched

Medline OVID Racial trauma - Rac* adj4 (stress or trauma or distress) OR "ethno-racial trauma" Population - Black or "African Canadian" or "African American" or "Afro-Caribbean" or “Afro-Canadian” Conceptualization- Concept* OR Defin* OR terminology OR manifestation OR classif* "1 exp Racism/ 8483

2 Racial Groups/ 27886

3 ""trauma and stressor related disorders""/ or exp stress disorders, traumatic/ 50761

4 1 or 2 35719

5 3 and 4 200

6 (Rac* adj4 (stress or trauma or distress)).ti,ab,kw. 2318

7 ""ethno-racial trauma"".ti,ab,kw. 5

8 5 or 6 or 7 2446

9 (Black or ""African Canadian"" or ""African American"" or ""Afro-Caribbean"" or ""Afro-Canadian"").ab,ti,kw. 247318

10 exp Black People/ 103140

11 9 or 10 289218

12 (Concept* or Defin* or terminology or manifestation or classif*).ti,ab,kw. 3197919

13 8 and 11 and 12 155

14 limit 13 to yr=""2000 -Current"" 148

" 148 June 24th, 2025

APA PsycINFO OVID same as above "1 (Rac* adj4 (stress or trauma or distress)).ab,id,ti. 2841

2 ""ethno-racial trauma"".ab,id,ti. 6

3 exp racial trauma/ 259

4 exp racism/ 13152

5 exp trauma/ 93795

6 4 and 5 494

7 1 or 2 or 3 or 6 3066

8 (Black or ""African Canadian"" or ""African American"" or ""Afro-Caribbean"" or ""Afro-Canadian"").ab,id,ti. 121802

9 exp black people/ 66333

10 8 or 9 128587

11 (Concept* or Defin* or terminology or manifestation or classif*).ab,id,ti. 933585

12 7 and 10 and 11 280

13 limit 12 to yr=""2000 -Current"" 267

" 267 June 24th, 2025

Anthropology Plus 0

CINAHL ( (MH "Racialization") OR (MH "Racism+") ) AND (MH "Trauma") OR XB ( Rac* N4 (stress or trauma or distress) ) OR XB "ethno-racial trauma" AND XB (Black or "African Canadian" or "African American" or "Afro-Caribbean" or “Afro-Canadian”) OR (MH "Black Persons+") AND XB (Concept* OR Defin* OR terminology OR manifestation OR classif*) 120 June 24th, 2025

Public Health Database (noft(Rac* NEAR/4 (stress OR trauma OR distress)) OR noft("ethno-racial trauma")) AND noft(Black OR "African Canadian" OR "African American" OR "Afro-Caribbean" OR "Afro-Canadian") AND noft(Concept* OR Defin* OR terminology OR manifestation OR classif*) 59 June 24th, 2025

Web of Science Core Collection (((AB=(Rac* NEAR/4 (stress or trauma or distress))) OR AB=("ethno-racial trauma" )) AND AB=( Black or "African Canadian" or "African American" or "Afro-Caribbean" or “Afro-Canadian” )) AND AB=(Concept* OR Defin* OR terminology OR manifestation OR classif* ) 204 June 24th, 2025

SCOPUS TITLE-ABS-KEY ( ( Rac* W/ 4 ( stress OR trauma OR distress ) ) OR "ethno-racial trauma" AND Black OR "African Canadian" OR "African American" OR "Afro-Caribbean" OR "Afro-Canadian" AND Concept* OR Defin* OR terminology OR manifestation OR classif* ) 326 June 24th, 2025

Sociological Abstracts (noft(Rac* NEAR/4 (stress OR trauma OR distress)) OR noft("ethno-racial trauma")) AND noft(Black OR "African Canadian" OR "African American" OR "Afro-Caribbean" OR "Afro-Canadian") AND noft(Concept* OR Defin* OR terminology OR manifestation OR classif*) 99 June 24th, 2025

PTSDpubs (noft(Rac* NEAR/4 (stress OR trauma OR distress)) OR noft("ethno-racial trauma")) AND noft(Black OR "African Canadian" OR "African American" OR "Afro-Caribbean" OR "Afro-Canadian") AND noft(Concept* OR Defin* OR terminology OR manifestation OR classif*) 6 June 24th, 2025

ERIC OVID "1 (Rac* adj4 (stress or trauma or distress)).ti,id,ab. 401

2 ""ethno-racial trauma"".ti,id,ab. 1

3 exp racism/ 5336

4 exp race/ 9954

5 trauma/ 3323

6 3 or 4 14823

7 5 and 6 159

8 1 or 2 or 7 512

9 (Black or ""African Canadian"" or ""African American"" or ""Afro-Caribbean"" or ""Afro-Canadian"").ti,id,ab. 52986

10 Blacks/ or African Americans/ 20210

11 9 or 10 58951

12 (Concept* or Defin* or terminology or manifestation or classif*).ti,id,ab. 328406

13 8 and 11 and 12 43

14 limit 13 to yr=""2000 -Current"" 39

" 39 June 24th, 2025
